# Effects of common Gram-negative pathogens causing male genitourinary-tract infections on human sperm functions

**DOI:** 10.1038/s41598-021-98710-5

**Published:** 2021-09-28

**Authors:** Sara Marchiani, Ilaria Baccani, Lara Tamburrino, Giorgio Mattiuz, Sabrina Nicolò, Chiara Bonaiuto, Carmen Panico, Linda Vignozzi, Alberto Antonelli, Gian Maria Rossolini, Maria Torcia, Elisabetta Baldi

**Affiliations:** 1 Andrology, Women’s Endocrinology and Gender Incongruence Unit, Careggi Hospital, 50139 Florence, Italy; 2 Clinical Microbiology and Virology Unit, Florence Careggi University Hospital, 50139 Florence, Italy; 3 Department of Experimental and Clinical Medicine, University of Florence, 50134 Florence, Italy; 4 Department of Experimental and Clinical Biomedical Sciences “Mario Serio”, University of Florence, 50134 Florence, Italy

**Keywords:** Molecular medicine, Endocrinology

## Abstract

Male genitourinary tract (MGT) bacterial infections are considered responsible for 15% of male infertility, but the mechanisms underlying decreased semen quality are poorly known. We evaluated in vitro the effect of strains of Gram-negative uropathogenic species (two *E.coli* strains, three *K. pneumoniae* strains, *P. aeruginosa* and *E. cloacae*) on motility, viability, mitochondrial oxidative status, DNA fragmentation and caspase activity of human spermatozoa. All strains, except *P. aeruginosa*, reduced significantly sperm motility, with variable effects. Sperm Immobilizing Factor (SIF) was largely responsible for deteriorating effects on sperm motility of *E. coli* strains since they were completely reverted by knockout of SIF coding *recX* gene. Sequence alignment for RecX showed the presence of high homologous sequences in *K. pneumoniae* and *E. cloacae* but not in *P. aeruginosa.* These results suggest that, in addition to *E.coli*, other common uropathogenic Gram-negative bacteria affect sperm motility through RecX products. In addition to sperm motility, the *E. coli* strain ATCC 35218 also affected sperm viability, and induced caspase activity, oxidative stress and DNA fragmentation suggesting an interspecies variability in the amount and/or type of the produced spermatotoxic factors. In general, our results highlight the need for a careful evaluation of semen infections in the diagnostic process of the infertile man.

## Introduction

Male factor is responsible of 40–50% of couple infertility, and it is estimated that male infertility affects up to 15% of the couples^1^. The most common cause of male infertility is poor semen quality, which may be due to alterations of testicular function or may originate during sperm transit in the male genital tract. Acute and chronic inflammation and infections are believed to be responsible for approximately 15% of cases of male infertility likely because of a detrimental effect on spermatozoa, although the association between inflammation and infections and poor semen quality has not been clearly defined^2^. *Enterobacterales* spp. are common pathogens of the urogenital tract and may interfere with male fertility^3,4^. As reported in the study by Boeri et al.^5^, *Enterobacteriaceae* represent the second most frequent pathogens responsible for semen infections in a cohort of 1689 European male partners of primary infertility couples. Similar frequencies were found in subfertile men attending the outpatient clinic of the University hospital of Florence^6^.

*Escherichia coli* is one of the most frequent species found in human semen^6,7^ and in genitourinary infections^8^, in particular epididymitis^9^. *E. coli* rapidly adheres to human spermatozoa in vitro, resulting in agglutination of spermatozoa. A profound decline in motility of spermatozoa is evident over time caused by severe alterations in sperm morphology^7^ and by the release of soluble spermatotoxic factors such as sperm immobilizing factor (SIF,^2^). An association with oligoasthenozoospermia and male infertility was however reported also with other *Enterobacteriaceae* as *Klebsiella* p*neumoniae* and *Klebsiella aerogenes*^10^ and with *Pseudomonas aeruginosa*^5^.

Evaluation of the direct effects of bacteria on sperm functions in vitro is of great help in understanding the role of infections in male infertility. So far, most in vitro studies evaluating the effects of *Enterobacteriaceae* on human spermatozoa, employed *E. coli* strains as pathogen (for review see^2,8^). In addition, most studies were limited to evaluate the effect of *E. coli* on human sperm motility and viability, and have been performed on highly motile selected sperm populations^11–15^, which are poorly representative of the real environment where bacteria, present in the male genital tract, may produce the damage. Whether other bacterial species as *K. pneumoniae*, *K. aerogenes*, *Enterobacter cloacae*, *P. aeruginosa,* commonly causing genitourinary tract infections (GUTI), affect human sperm motility or other sperm functions is not yet known. Although progressive motility and sperm viability are of fundamental importance both in natural and assisted reproduction, other sperm characteristics are necessary for fertilization and embryo development. In particular, spermatozoa must deliver an intact DNA to the oocyte. Oxidative and apoptotic pathways may cause sperm DNA fragmentation (sDF;^16,17^), the most common type of DNA damage found in human spermatozoa^18^, which has a negative impact on both natural and assisted reproduction^19^. The effects of bacteria on sperm oxidative and apoptotic pathways have been poorly investigated. Besides damaging sperm DNA by inducing fragmentation, base oxidation and mutations, oxidative stress, when present in high levels, can cause lipid peroxidation in the plasma membrane, produce modifications of sperm proteins impairing their functions, alter mitochondrial function and induce apoptosis^20^. In turn, activation of apoptotic pathways may impact on other sperm functions and, ultimately, leading to cell death^21^.

Inhibitory effect of *E. coli* on sperm motility have been attributed to release of SIF^2,8^, however, whether SIF is involved in the inhibitory effect of other bacterial species is presently less clear, nor it is known the role of this factor in other sperm alterations due to bacterial infections.

In the present study we selected bacterial strains belonging to potential uropathogenic species (*E. coli*, *K. pneumoniae*, *K. aerogenes *and *E. cloacae)* that express the *rec-X* SIF-coding gene or highly homologous genomic sequences and evaluated their effects on sperm motility, viability, mitochondrial oxidative status, DNA fragmentation and caspase activity in whole semen.

*P. aeruginosa*, a less frequent pathogen of male urogenital apparatus which was found with a prevalence of 10% in infertile couples^22^ was also included in the study.

In addition, we further explored the role of SIF on sperm motility by using an *E. coli* strain KO for SIF coding gene.

## Results

### Effect of bacterial strains on sperm motility

**Figure 1:**
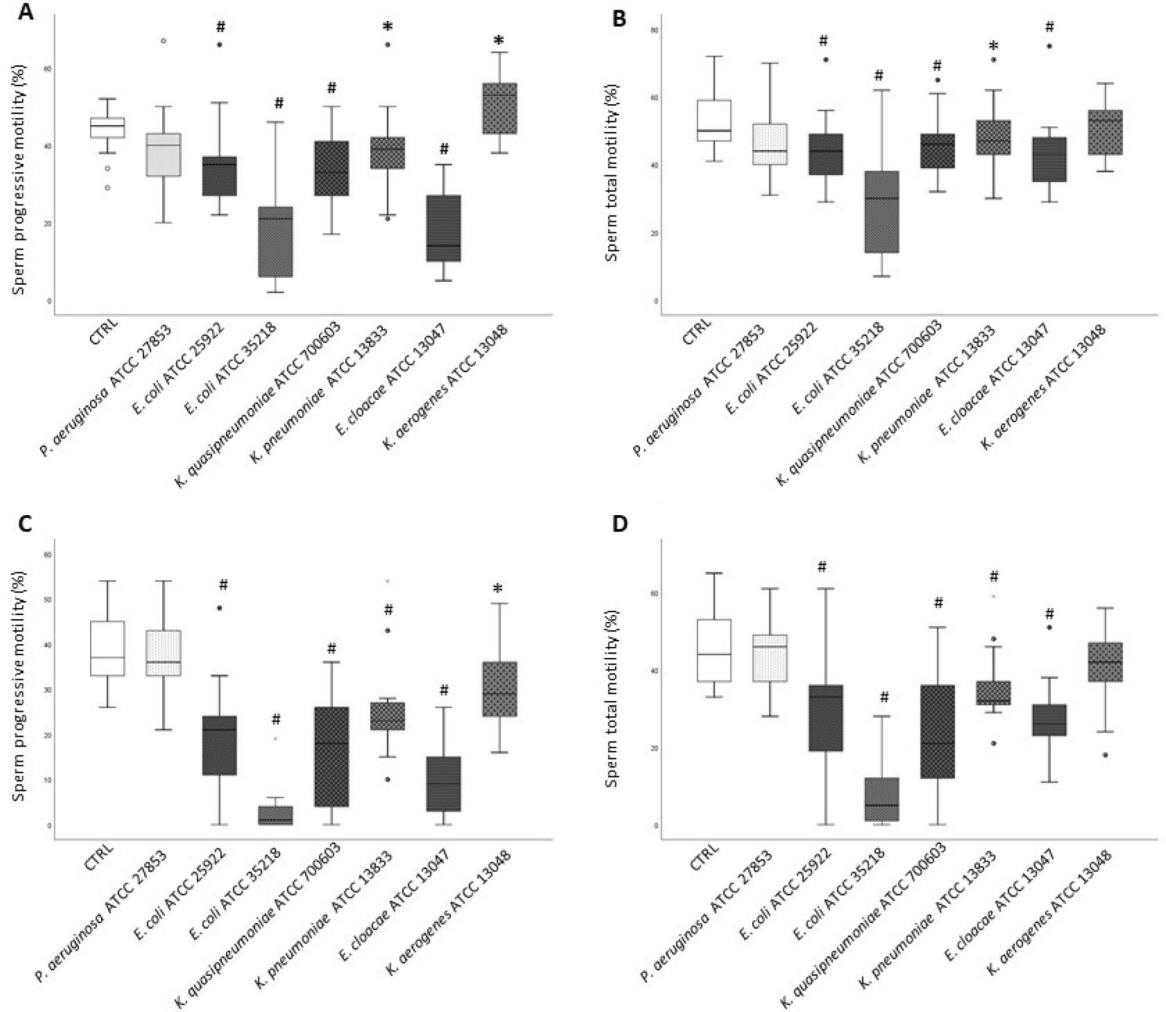
Box-plots representing the effect of 1 (n = 13, panels **A** and **B**) and 3 (n = 16, panels **C** and **D**) hours in vitro incubation (37 °C, 5%CO_2_) of whole semen samples with various bacterial strains (*P. aeruginosa* ATCC 27853, *E. coli* ATCC 25922, *E. coli* ATCC 35218, *K. quasipneumoniae* ATCC 700603, *K. pneumoniae* ATCC 13883, *E. cloacae* ATCC 13047 and *K. aerogenes* ATCC 13048) on sperm progressive (**A** and **C**) and total motility (**B** and **D**). Wilcoxon test, *p < 0.05; #p < 0.01 versus CTRL.

Whole semen samples were incubated with live bacterial cells from *E. coli* (ATCC 29522 and ATCC 35218), *K. pneumoniae*, *P. aeruginosa*, *E. cloacae* and *K. aerogenes* strains at sperm/bacteria ratio of 1:10^23^ and progressive and total motility was recorded after 1 (n = 13) and 3 h (n = 16). All bacteria strains, except *P*. *aeruginosa* ATCC 27853, determined a significant decrease of total and progressive motility both at 1 (Fig. 1, panels A and B) and 3 (Fig. 1, panels C and D) hours incubations. Among the bacteria tested, *E. cloacae* ATCC 13047 and *E. coli* ATCC 35218 strain were the most potent in reducing sperm progressive motility at both time points. A significant increase in the percentage of non-progressive motility of spermatozoa was detected in cultures with *K. quasipneumoniae* ATCC 700603, *K. aerogenes* ATCC 13048 and *E. cloacae* ATCC 13047 strains (data not shown). A direct adhesion of bacterial cells to the sperm tails as well as sperm agglutination was observed in cultures with *E. cloacae* ATCC 13047 (Supplemental Figure S1 and video S1), whereas such effects were not observed with the other bacterial strains (data not shown).

### Role of SIF on sperm motility

The 56 kDa Sperm Immobilizing Factor (SIF), is considered one of the responsible factors of the detrimental effects of *E. coli* on sperm motility^24^.

**Figure 2:**
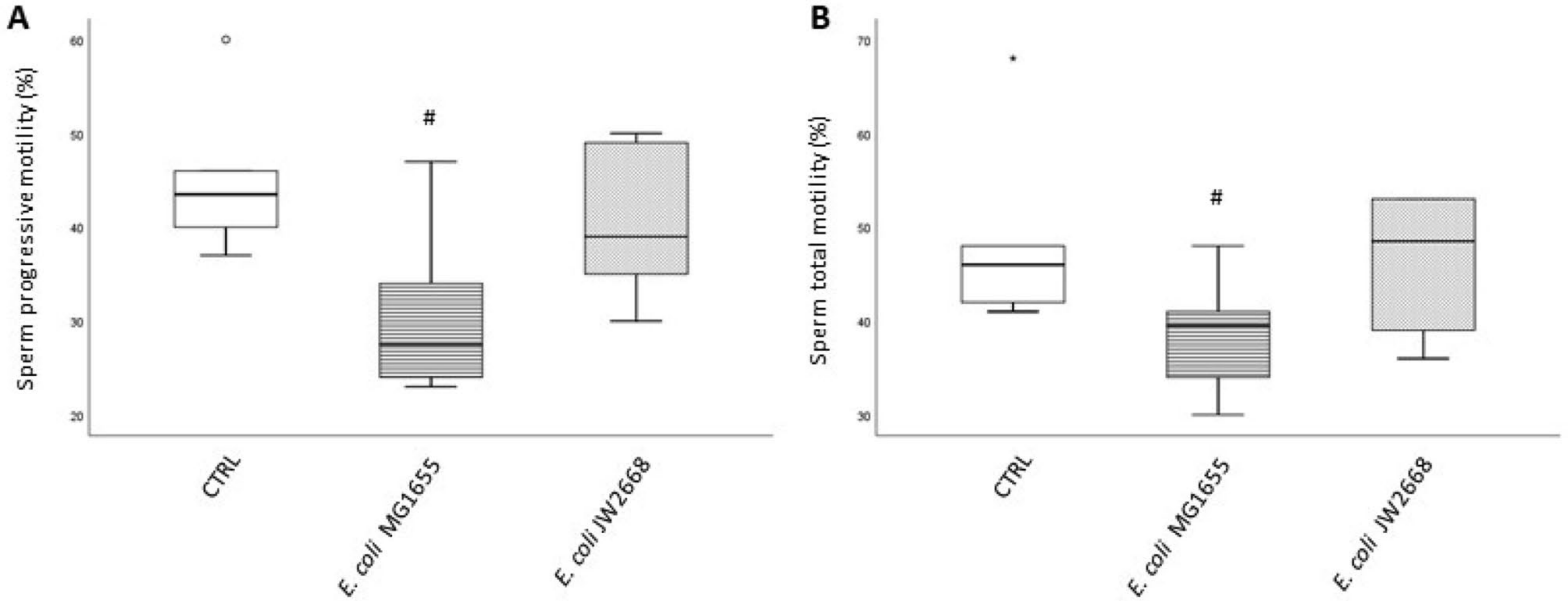
Box-plots representing the effect of 3 h in vitro incubation (37 °C, 5%CO_2_) of whole semen samples (n = 6) with *E. coli* MG1655 (wild-type strain) and *E. coli* JW2668 (knockout for SIF) on sperm progressive (A) and total (B) motility. Wilcoxon test, *p < 0.05; #p < 0.01 versus CTRL.

To further confirm the role of SIF in sperm motility, we performed experiments by using the *E. coli* MG1655 as reference strain and its mutated counterpart JW2668 knockout (KO) for *recX* gene^25^. JW2668 *E. coli* strain did not affect either progressive (Fig. 2A) and total (Fig. 2B) sperm motility after 3 h incubation whereas the wild type MG1655 strain decreased both motilities.

**Figure 3:**
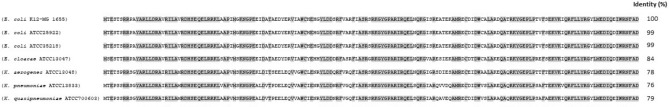
Amino acid sequence alignment of homologous RecX proteins. Conserved amino acidic regions are shaded in grey. Percentage of aminoacidic sequence identity of SIF homologous among *Enterobacterales* strains using *E. coli* MG1655 as reference strain was also reported.

To investigate whether also the other strains affecting sperm motility were able to produce SIF homologous proteins, we performed tBLASTn and BLASTp sequence alignments using *recX* from *E. coli* K12 MG1655 as reference sequence. The results of this analysis, shown in Fig. 3, clearly indicate a high homology sequence in *recX* gene and SIF protein among the strains of *E. coli* (both strains), *K. pneumoniae* (both strains), *K. aerogenes* and *E. cloacae*. Conversely, sequence homology with RecX was not detected in the genome of *P. aeruginosa* ATCC 27853 (not shown), an *Enterobacteriales* species which belongs to the *Pseudomonacee* family.

### Effect of bacterial strains on sperm viability

**Figure 4:**
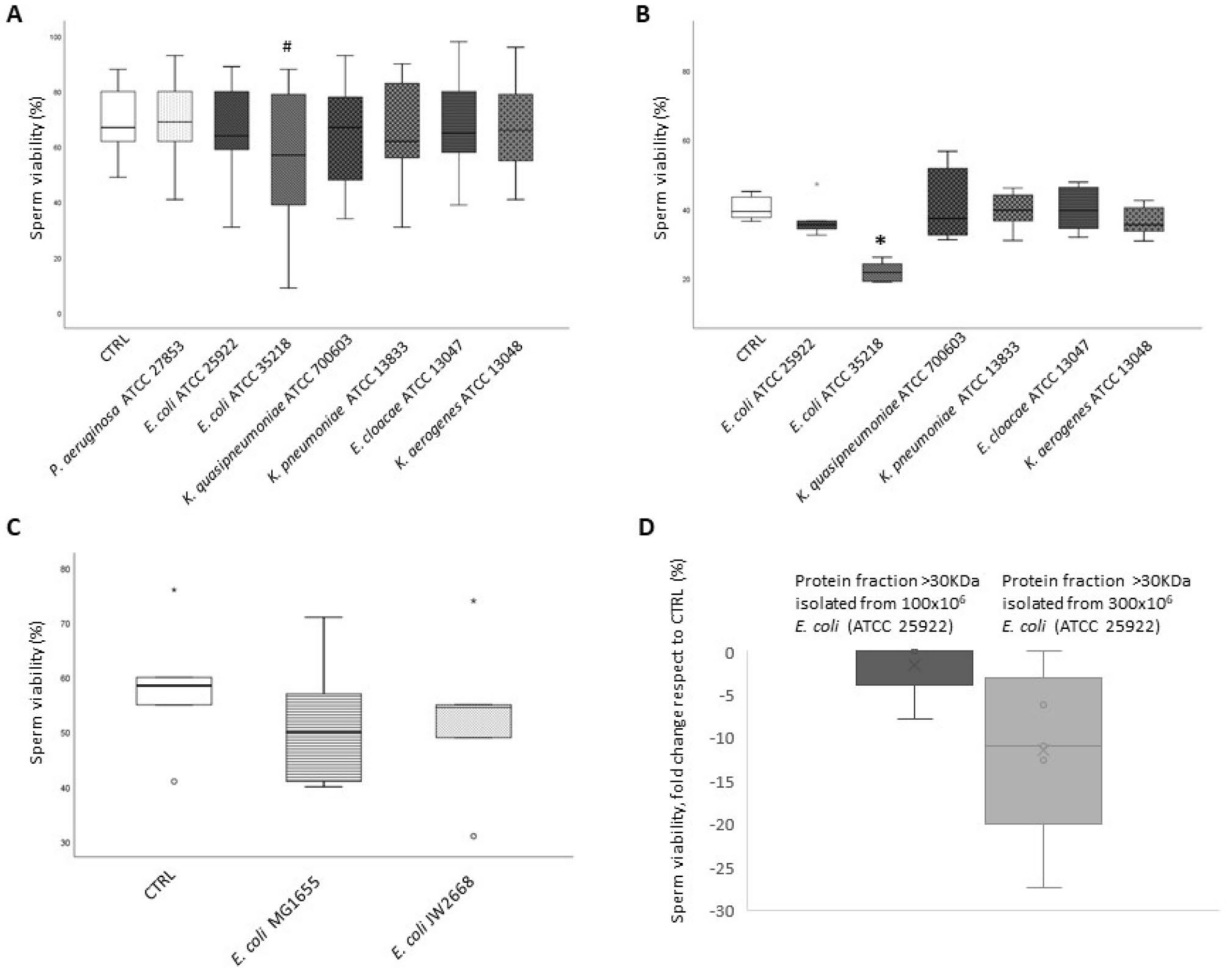
Box-plots representing the effect of 3 h in vitro incubation (37 °C, 5%CO_2_) of whole semen samples with various bacterial strains (*P. aeruginosa* ATCC 27853*, E. coli* ATCC 25922*, E. coli* ATCC 35218*, K. quasipneumoniae* ATCC 700603*, K. pneumoniae* ATCC 13883*, E. cloacae* ATCC 13047 and *K. aerogenes* ATCC 13048) on sperm viability determined by eosin staining (n = 16, panels **A** and **C**) and by the LIVE/DEAD™ Fixable Green Dead Cell Stain (n = 6, panel **B**). In panel **C**, box-plots representing the effect of 3 h in vitro incubation (37 °C, 5%CO_2_) of whole semen samples (n = 6) with *E. coli MG1655* (wild-type strain) and *E. coli JW2668* (knockout for SIF) on sperm viability. In panel **D**, box plots representing sperm viability fold change respect to CTRL of 5 subjects after incubation with supernatants of *E. coli* ATCC 25922 obtained from a different number of cultured bacteria (100 and 300 × 10^6^), after isolating protein fractions ≥ 30 KDa. Wilcoxon test, *p < 0.05; #p < 0.01 versus CTRL.

To investigate whether changes in motility were due to a decrease of sperm viability, we evaluated the percentage of viable spermatozoa after 3 h of incubation with the different bacteria by two different techniques (eosin staining and Live⁄Dead Fixable Green Dead Cell Stain coupled to flow cytometry). Figure 4 shows that, with the exception of *E. coli* ATCC 35218, none of the bacterial strains induced significant reduction in cell viability with both methods (A, B). Similar results were obtained after 1 h incubation (not shown). Neither the reference *E. coli* MG1655 strain (wild-type strain), nor the *E. coli* JW2668 strain (KO for *recX* gene), affected significantly sperm viability as evaluated by eosin staining (Fig. 4C). Since SIF was reported to reduce sperm viability at higher concentrations respect to those affecting motility^24^, we collected supernatants from *E. coli* ATCC 25922 cultured at 100 and 300 × 10^6^/ml, and purified the fractions containing proteins with molecular weights (MW) ≥ 30KDa, thus including the 56 kDa SIF. After incubation for 3 h with such fractions, a decrease in sperm viability was observed with the fractions obtained from 300 million bacteria (Fig. 4D), suggesting that the toxic effect depends on the rate of secretion of spermatotoxic factors and that such rate is variable within strains of the same species.

### Effect of bacterial strains on sperm oxidative stress, caspase activity and DNA fragmentation

**Figure 5:**
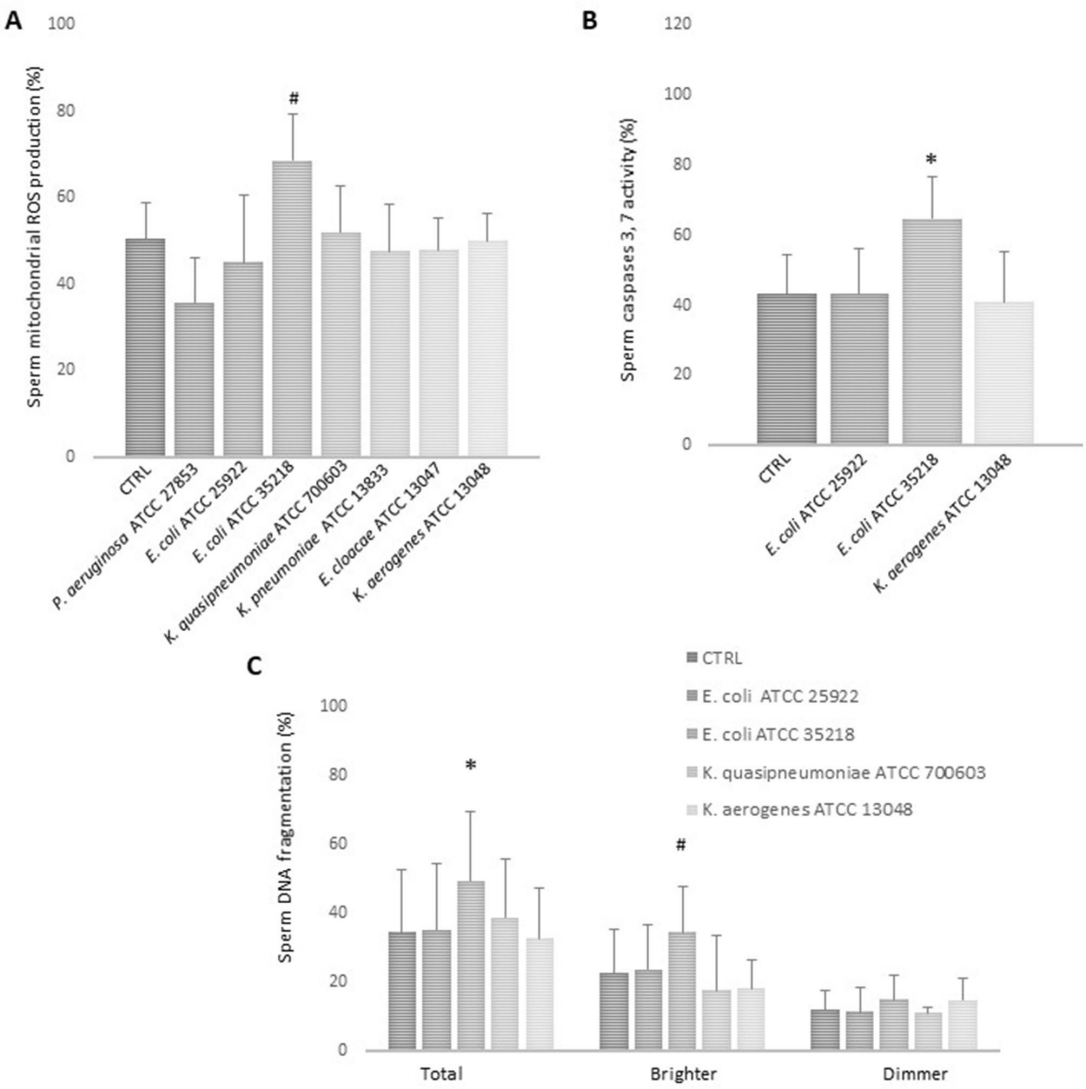
Histograms representing the effect of 3 h in vitro incubation (37 °C, 5%CO_2_) of whole semen samples on sperm mitochondrial ROS production, measured by Mitosox Red, with all tested bacterial strains (*P. aeruginosa* ATCC 27853, *E. coli* ATCC 25922, *E. coli* ATCC 35218, *K. quasipneumoniae* ATCC 700603, *K. pneumoniae* ATCC 13883, *E. cloacae* ATCC 13047 and *K. aerogenes* ATCC 13048) (n = 6, panel **A**), sperm caspases 3 and 7 activity, measured by FLICA, with *E. coli* ATCC 25922, *E. coli* ATCC 35218 and *K. aerogenes* ATCC 13048 (n = 5, panel **B**) and sperm DNA fragmentation, measured by TUNEL/PI, with *E. coli* ATCC 25922, *E. coli* ATCC 35218, *K. quasipneumoniae* ATCC 700603 and *K. aerogenes* ATCC 13048 (n = 5, panel **C**). Student t-test, *p < 0.05; #p < 0.01.

Pathogenic strains of *Enterobacteriaceae* family are known to produce toxins or metabolites that induce oxidative stress in infected cells^26^. In order to understand whether the bacterial strains tested in our study could determine an increase in ROS production and sperm oxidative status, we evaluated mitochondrial oxidation by using the fluorescent probe MitoSOX™ Red. With the exception of *E. coli* ATCC 35218, none of tested bacterial strains affected sperm mitochondrial ROS generation (Fig. 5A).

Interestingly, *E. coli* ATCC 35218 also affected sperm viability (Fig. 4 A and B), suggesting that generation of oxidative stress could be involved in inducing sperm death through activation of an apoptotic pathway^16^. To support this hypothesis, the activity of caspase 3 and caspase 7, an established marker of sperm apoptosis^27^, was measured. As shown in Fig. 5B, E*. coli* ATCC 35218 increased significantly the percentage of spermatozoa expressing caspase 3 and 7 activity. *E. coli* ATCC 25922 and *K. aerogenes*, used as control strain that did not affect sperm viability (Fig. 4 A and B) and mitochondrial oxidation (Fig. 5A), did not induce caspase activation. *E. coli* ATCC 35218 also induced a significant increase of total and PI brighter sDF^28^, whereas the other tested bacterial strains did not affect these parameters (Fig. 5C).

## Discussion

Although bacterial pathogens are frequently found in semen samples of infertile men, there is no consistent epidemiological link between pathogens and male infertility or altered semen parameters. However, a recent study^2^ reported that bacteriospermia was directly related to 15% of infertility in men treated with assisted reproduction. In our knowledge, the present study is the first one where the in vitro effect of a wide panel of bacteria belonging to the *Enterobacteriaceae* family commonly found in infected semen^5^, was evaluated at the same time and in the same semen samples. We found that almost all bacterial strains directly affect human sperm motility, whereas, only the *E. coli* strain ATCC 35218 impaired sperm viability, induced mitochondrial oxidative stress, DNA fragmentation and activate the apoptotic pathway. Even if the strains included in the study were not isolated from infertile males, *K. quasipneumoniae* ATCC 700603 (ST489), has been isolated from a urine sample, and *E. coli* ST73 and ST127 (the clones of ATCC 25922 and ATCC 35218, respectively) were recently associated to hospital and community acquired urinary tract infections^29,30^. In agreement with previous studies^11–14,31,32^ the reduction of sperm motility was observed following incubation in vitro with SIF-producing strains of *E. coli*. Our study extends these findings to other *Enterobacteriaceae*, whose in vitro effects on human spermatozoa have been less investigated. However, the detrimental effects on motility were highly variable and dependent on the strain.

We demonstrated the presence of high homologous sequences to *recX* gene and SIF protein from *E. coli* MG1655 in the *K. pneumoniae*, *K. aerogenes* and *E. cloacae* strains, suggesting that the secretion of this toxin may act as a common molecular mechanism used by *Enterobacteriaceae* to immobilize sperm. This conclusion is reinforced by the use of an *E. coli* strain KO for the *recX* gene that did not affect sperm motility compared to the wild type. In addition, *P. aeruginosa,* which is found in about 10% of infertile couples^22^ and belongs to a different taxonomic order (*Pseudomonadales*), does not express a homologous *recX* gene and does not have the property to immobilize sperm in vitro.

We noted that, despite the culture with *K. pneumoniae*, *K. aerogenes*, *E. cloacae* strains significantly decreased sperm progressive motility, such strains did not completely immobilize spermatozoa but increased the percentage of in situ motility. This result suggests that complete immobilization is influenced by the quality or the quantity of SIF released by bacterial strains. In addition, the *E. coli* strains used in our study did not determine sperm agglutination, which was observed only after incubation with *E. cloacae*.

The effects on sperm progressive and total motility were present already after 1 h incubation and did not vary substantially after 3 h for most bacterial strains, indicating that the effect on motility may be quite rapid. This result suggests that waiting long times before sperm manipulation in assisted reproduction laboratories or during routine semen analysis, may result in a decrease of sperm motility and of the number of sperm recovered after selection procedures when strains of *E. coli*, *K. pneumoniae*, *K. aerogenes*, *E. cloacae* are present in semen.

We show that most of the *Enterobacteriaceae* tested here reduce motility without affecting sperm viability, with the exception of the *E. coli* strain ATCC 35218. This result was confirmed by using two different methods to assess sperm viability, a subjective one (eosin staining) according to indications of WHO^33^ and an objective one (staining with LIVE/DEAD™ Fixable Green Dead Cell Stain coupled to flow cytometric detection). The results were qualitatively similar, although viability evaluated after staining with LIVE/DEAD™ Fixable Green Dead Cell Stain was found lower in all samples. It is possible that LIVE/DEAD™ Fixable Green Dead Cell Stain is more efficient than eosin in detecting unviable spermatozoa or is able to stain also apoptotic cells committed to die. We should also consider that the subjective analysis is done on 200 spermatozoa whereas the flow cytometric analysis regards 8.000 events, and thus, likely, more precise.

Unlike motility, the effect of bacteria on viability appears to be dependent from the amount of SIF released in culture. In fact, when spermatozoa were incubated with the SIF-containing purified fraction from 300 millions *E. coli* ATCC 25922 culture supernatants, a partial spermicidal effect was observed, in agreement with previous studies^24^. However, we cannot exclude that factors different from SIF, produced by *E. coli* ATCC 35218, could be also involved in viability impairment. Of note, our analysis revealed that different *E. coli* strains could have a spectrum of different effects on sperm functions going from decrease of motility to induction of oxidative stress, DNA damage and cell death. In particular, the *E. coli* ATCC 35218 was the only strain, among those tested, able to increase mitochondrial ROS production, activate apoptotic pathways and induce sperm DNA fragmentation.

Oxidative stress may be both a consequence and an inducer of sperm apoptosis^16,34^, and may be also involved in increasing DNA fragmentation^16,35^. In particular, oxidative stress appears to be the main inducer of sDF after spermiation and during in vitro incubations^16,36–38^.

The inducing effects of *E. coli* isolates on oxidative status and apoptosis were reported previously using different experimental conditions compared to our study^14,15,39^. In particular, Boguen et al.^15^, by comparing three *E. coli* strains demonstrated that the hemoliytic strain shows a greater detrimental effect on spermatozoa respect to non-haemolytic ones, including the *E. coli* strain ATCC 25922. A comparative genomic analysis of *E. coli* strains used in our study revealed the unique presence in *E. coli* ATCC 35218 of the chromosomal HlyE gene coding for hemolysinE (data not shown), a toxin with a short half-life that is known to impair membrane integrity in other cell types^40^. Therefore, it is possible that the detrimental effects of *E. coli* strain ATCC 35218 are mediated by more than one spermotoxic factor^41^.

Reduction of sperm viability and motility, as it may occur in the case of semen infections, may highly affect the reproductive performance both in natural and assisted conception, as progressive motility is the necessary pre-requisite to reach the oocyte and to penetrate its vestments, whereas viability is of fundamental importance for a correct fertilization. In particular, motility is the primary sign used to determine sperm viability during intracytoplasmic sperm injection (ICSI). If no motility is present in a sample, techniques to identify viable spermatozoa can be used by embryologists^42^. In case of *E. coli* ATCC 35218, where reduction of motility may exceed reduction of viability (89% vs 45% according to viability evaluated by LIVE/DEAD™ Fixable Green Dead Cell Stain), viable spermatozoa may show increased oxidative stress and/or activation of apoptotic pathway and/or fragmented DNA, likely compromising the outcome of reproduction. In particular, the *E. coli* ATCC 35218 induces DNA fragmentation within the PI brighter sperm population, which is unrelated to semen quality and may contain viable DNA fragmented spermatozoa^28^. sDF is associated with a reduced performance in ART affecting implantation and increasing the probability of miscarriage^43–46^. The effect of bacterial contamination in semen on the outcomes of ARTs is controversial. Some studies indicated poor outcomes because of oocyte degeneration^47,48^ whereas others did not report significant effects on ART outcomes^49^. Incubation in vitro with *E. coli* reduces sperm ability to penetrate hamster oocytes, suggesting a negative effect on fertilization ability^32^.

A strength of our study is testing the effect of strains of the most frequent *Enterobacteriaceae* infecting male reproductive tract, evidencing differences in their effects on sperm characteristics. In addition, we evaluated the effect of bacteria in the natural environment where they may alter sperm functions. In contrast, most previous studies have been performed on highly motile selected sperm^11–15^ or washed semen samples^39^, where the effect of bacteria is tested in a medium where they never act, and that does not contain substances that can limit or enhance their effects. For instance, it has been shown lactobacilli^14^ may prevent the effect of *E. coli* on sperm motility. In addition, fragmented semenogelins generated after liquefaction^50^ and enzymes present in semen^51^ show antibacterial activity. A limitation of our study is represented by the use of commercially available bacterial strains and not those isolated from semen samples. Moreover, we chose to use strains of *Enterobacteriaceae* with known genome to allow the sequence alignment shown in Fig. 3. Such alignment allowed us to reveal that other *Enterobacteriaceae* contain *recX* homologous sequences in their genome.

In conclusion, our data indicate that common uropathogenic Gram-negative bacteria induce an impairment of sperm motility through *recX* products and suggest that an increased secretion of SIF or other factors, produced by selected strains, may be involved in impairing other sperm functions. Since the effects of bacteria on human spermatozoa may be variable and dependent on the strain, a careful evaluation of semen infections in the diagnostic process of the infertile man is warranted. Further experiments performed on bacterial samples isolated from semen cultures will be necessary in order to reinforce experimental proofs of SIF homologous activity secreted by *Enterobacteriaceae* strains derived from their natural site of infection.

## Materials and methods

### Ethic statement

The study was approved by the local Ethical Committee Comitato Etico Area Vasta Centro (CEAVC, protocol n. 16764_bio). All research was performed in accordance with the Declaration of Helsinki. Patients were informed about the aim of the study and signed an informed consent to use the remaining semen after routine analysis.

### Reagents and bacteria

Human tubal fluid (HTF) medium was purchased from Biocare Europe (Rome, Italy). MitoSOX™ Red, LIVE/DEAD™ Fixable Green Dead Cell Stain and Vybrant FAM Caspase 3 and 7 Assay Kit were purchased from Thermo Fisher Scientific (Waltham, MA, USA). In Situ Cell Death Detection Kit, fluorescein was obtained from Sigma Aldrich (St. Louis, MO, USA).

Seven bacterial reference strains were included in the study as follows: two isolates of *E. coli* (ATCC 29522 and ATCC 35218), one isolate of *K. pneumoniae* (ATCC 13883), one isolate of K. *quasipneumoniae* (ATCC 700603), one isolate of *P. aeruginosa* (ATCC 27853), one *E. cloacae* (ATCC 13047) and one *K. aerogenes* (ATCC 13048). An *E. coli* K12 strain MG1655 and its derivative (with the knockout *recX* gene), from the Keio collection, were also added to the study collection^52^. Most of the selected reference strains were isolated from clinical human samples, except for *K. pneumoniae* ATCC 13883 and *E. coli* ATCC 25922 whose source is unknown (Supplemental Table S1). All bacterial strains were seeded on CHROMID® CPS® Elite agar (bioMérieux, Marcy l’Etoile, France) and incubated for 18 h at 35 ± 1 °C. Bacterial suspensions were prepared in 2 ml of sterile water and optical density was measured by DensiCHEK™ spectrophotometer (bioMérieux).

### Extraction of proteins from bacterial supernatants

100 or 300 × 10^6^ *E. coli* cells from ATCC 25922 strain were cultured in Mueller Hinton broth at 37 °C overnight. Culture supernatants were collected and centrifuged at 4000 g for 10 min. The protein fraction with MW ≥ 30 kDa was purified using Centrifugal Filter Units (cut off 30,000 NWML) (Amicon® Ultra-4 and -15 Centrifugal Filter Units – 30,000 NMWL, Merk, Darmstadt, Germany) according to manufacturing recommendations. Protein amount in the purified fraction was quantified by BCA (Bicinchoninic Acid) method and used at 15 and 45 µg/µL.

### Sequence alignment

Reference strains genome were downloaded from LGC website (www.lgcstandards-atcc.org), database homology searches of proteins were carried out using tBLASTn and BLASTp (https://blast.ncbi.nlm.nih.gov/Blast.cgi) software and amino acidic sequences alignments were performed by AlignX (Invitrogen, Carlsbad, USA) using *E. coli* K12 MG1655 as consensus sequence.

### Sperm samples and processing

Semen samples were obtained by masturbation from patients undergoing routine semen analysis for couple infertility, in the Andrology laboratory of the University of Florence, Italy. Semen analysis was carried out according to World Health Organization (WHO) guidelines^33^. Semen samples with leukocytes and/or evident bacteria were excluded from the study. For the study purpose, spermatozoa from n = 32 normozoospermic subjects (see Supplemental Table S2 for semen characteristics) were included. After counting of spermatozoa, whole semen samples were divided in 9 equal aliquots and seeded in 96-well plates at concentration range between 1 × 10^6^ and 10 × 10^6^ cells per well in a final volume of 100 µl. The bacteria infection assay was performed by incubating spermatozoa in presence of bacterial strains at 1, 10 and 100 MOI/cell for 1 and 3 h at 37 °C in a humidified chamber with 5% CO_2_. The maximum effect was reached at 10 MOI/cell (data not shown). 10 MOI/cell was then used for all the experiments shown. An equal volume of sterile water was added to control aliquots.

### Evaluation of sperm motility

After incubation with different bacterial strains, the percentage of progressive and total sperm motility where checked at the optical microscopy, according to WHO criteria^33^, evaluating at least 200 spermatozoa for each experimental point. The analysis was conducted in the Laboratory of Andrology of the Florence Careggi University Hospital that participates in the UK-NEQAS (United Kingdom National External Quality Assessment Service) external quality control program for semen analysis since 2005. The mean (± sd) percent biases of the laboratory for the years 2019 were 7.0 (± 15.6) and 1.1 (± 11.2), respectively, for total and progressive motility and 9.2 (± 6.7) for sperm concentration (n = 16, data from UK-NEQAS).

### Evaluation of sperm viability

Sperm viability was evaluated by using eosin staining^33^ and by LIVE/DEAD™ Fixable Green Dead Cell Stain Kit. For eosin staining, sperm suspension and eosin (1%) (1:1) were mixed and then evaluated by optical microscopy assessing at least 200 spermatozoa for each aliquot. For staining with LIVE/DEAD™ Fixable Green Dead Cell Stain, after washing with HTF medium, semen samples were incubated for 1 h at room temperature, in the dark, in 500 μL of phosphate-buffered saline (PBS) with Live⁄Dead Fixable Green Dead Cell Stain Kit (diluted 1:50,000). Then samples were washed twice in PBS and acquired by flow cytometry (see below).

### Assessment of mitochondrial ROS generation

Mitochondrial ROS generation was evaluated using MitoSOX Red^14,53^ which shows distinct specificities toward superoxide^53^. After incubation with the different bacterial strains, spermatozoa were washed in PBS and then divided into two aliquots, one aliquot was re-suspended in 100 µL PBS (negative control) and one in 100 µL PBS containing Mitosox Red at a final concentration of 2 μM (test sample), and incubated for 15 min at room temperature. After wash in PBS, sperm samples were analysed by flow cytometry (see below).

### Evaluation of caspase activity

Caspases activity was evaluated by using Vybrant FAM Caspase-3 and -7 Assay Kit based on a fluorescent inhibitor of caspases (FLICA^TM^) according to Marchiani et al.^54^. After incubation with bacteria, each sample was splitted into two aliquots: a test sample re-suspended in 300 µL of PBS added with 10 μL of 30X FLICA working solution and a negative control incubated only with PBS. After 1 h incubation at 37 °C, samples were washed with Wash Buffer 1X and fixed with 40 μL of 10% formaldehyde for 10 min at room temperature. Wash and fixative solutions were supplied by the kit. Sperm samples were washed again twice and re-suspended in 400 µL of Wash Buffer 1X containing 6 µL of Propidium Iodide solution (PI, 50 µg/mL in PBS) and acquired by flow cytometry (see below).

### Evaluation of sperm DNA fragmentation

Sperm DNA fragmentation was evaluated by Tunel/PI method^28^. Briefly, after incubation with bacteria and washing twice with HTF medium, each aliquot was fixed with 200 µL of paraformaldehyde (4% in PBS, pH 7.4) for 30 min at room temperature. Semen samples were washed twice with 200 µL of PBS/1% bovine serum albumin (BSA), and then permeabilized with 100 µL of 0.1% sodium citrate/0.1% Triton X-100 (4 min in ice). Each sperm sample was divided into two aliquots and labelled with 50 µL of labelling solution (supplied by the kit) containing (test sample) or not (negative control) the terminal deoxynucleotidyl transferase (TdT) enzyme and incubated for 1 h at 37 °C in the dark. Samples were then washed twice, re-suspended in 500 µL of PBS and stained with 7.5 µL of PI (50 mg/mL, 10 min at room temperature in the dark) and acquired by flow cytometry.

### Flow cytometric analysis

Samples were acquired by a FACScan flow cytometer equipped with a 15-mW argon-ion laser for excitation. FL-1 (515–555-nm wavelength band) and FL-2 (563–607-nm wavelength band) detectors revealed green fluorescence of LIVE/DEAD™ Fixable Green Dead Cell Stain, caspases and Tunel and red fluorescence of Mitosox Red and PI, respectively. In the characteristic forward scatter/side scatter region of spermatozoa^28^, 8000 events were acquired. In the dot plot of fluorescence distribution of the negative sample, a marker, including 99% of total events, was established and translated in the corresponding test sample and all the events beyond the marker were considered positive. sDF was evaluated in the two sperm populations denominated PI brighter and PI dimmer^28^ and reported as percentage of sDF in the two populations and in total sperm. For acquisition and analysis, CellQuest-Pro software program (Becton–Dickinson) was used.

### Statistical analysis

Statistical analysis was performed using the Statistical package for the Social Sciences version 26.0 (SPSS, Chicago, IL, USA) for Windows. Data distribution was verified by using the Kolmogorov–Smirnov test. Data normally distributed were expressed as mean (± SD), whereas, data non-normally distributed as median (interquartile, IQR). Differences between groups were evaluated by paired two-sided Student’s t-test for normally distributed parameters, or by Wilcoxon signed-rank test for non-normally distributed parameters. A P-value of 0.05 was considered significant.

## Supplementary Information

Supplementary Legends.

Supplementary Video 1.

Supplementary Table 1.

Supplementary Table 2.

Supplementary Figure 1.
