# Author Correction: Effects of common Gram-negative pathogens causing male genitourinary-tract infections on human sperm functions

**DOI:** 10.1038/s41598-021-99771-2

**Published:** 2021-10-07

**Authors:** Sara Marchiani, Ilaria Baccani, Lara Tamburrino, Giorgio Mattiuz, Sabrina Nicolò, Chiara Bonaiuto, Carmen Panico, Linda Vignozzi, Alberto Antonelli, Gian Maria Rossolini, Maria Torcia, Elisabetta Baldi

**Affiliations:** 1 Andrology, Women’s Endocrinology and Gender Incongruence Unit, Careggi Hospital, 50139 Florence, Italy; 2 Clinical Microbiology and Virology Unit, Florence Careggi University Hospital, 50139 Florence, Italy; 3 Department of Experimental and Clinical Medicine, University of Florence, 50134 Florence, Italy; 4 Department of Experimental and Clinical Biomedical Sciences “Mario Serio”, University of Florence, 50134 Florence, Italy

Correction to: *Scientific Reports* 10.1038/s41598-021-98710-5, published online 28 September 2021

The original version of this Article contained an error in the spelling of the author Gian Maria Rossolini which was incorrectly given as Gianmaria Rossolini. The original Article and accompanying Supplementary Information files have been corrected.

