# Supplementary material for: Effects of common Gram-negative pathogens causing male genitourinary-tract infections on human sperm functions
Source: Sci Rep. 2021 Sep 28;11:19177. doi: 10.1038/s41598-021-98710-5 (PMC8478950; doi:10.1038/s41598-021-98710-5)
Supplement: Supplementary file 1 — Supplementary Legends. [file 41598_2021_98710_MOESM1_ESM.docx]

**Supplementary information legends**

**Supplemental Figure S1**. Frames from a video of a semen sample in vitro incubated with E. cloacae ATCC 13047 for 3 hours at 37°C, 5%CO2. Adhesion of bacteria to the tail is evident in many spermatozoa (Nikon Eclipse Ci phase contrast microscope with heated stage, Leica MC170 HD camera, resolution 5 Megapixel).

**Supplemental Video S1**. Video of a semen sample after in vitro incubation with E. cloacae ATCC 13047 for 3 hours at 37°C, 5%CO2 showing sperm agglutination and direct adhesion of bacteria to the sperm tail (Nikon Eclipse Ci phase contrast microscope with heated stage, Leica MC170 HD camera, resolution 5 Megapixel).
