# Supplementary material for: Effects of common Gram-negative pathogens causing male genitourinary-tract infections on human sperm functions
Source: Sci Rep. 2021 Sep 28;11:19177. doi: 10.1038/s41598-021-98710-5 (PMC8478950; doi:10.1038/s41598-021-98710-5)
Supplement: Supplementary file 3 — Supplementary Table 1. [file 41598_2021_98710_MOESM3_ESM.pdf]

# EFFECTS OF COMMON GRAM-NEGATIVE PATHOGENS CAUSING MALE GENITOURINARY-TRACT INFECTIONS ON HUMAN SPERM FUNCTIONS

Sara Marchiani, Ilaria Baccani, Lara Tamburrino, Giorgio Mattiuz, Sabrina Nicolò, Chiara Bonaiuto, Carmen Panico, Linda Vignozzi, Alberto Antonelli, Gian Maria Rossolini, Maria Torcia, Elisabetta Baldi

**Supplemental table 1.** Semen and age characteristics (expressed as median values [interquartile range]) of the subjects (n=32) recruited for the study.

| Number of subjects | Age (years) | pH | Volume (ml) | Concentration ($x10^6$/ml) | Number ($x10^6$/ejaculate) | Progressive motility (%) | Non-Progressive motility (%) | Immotile (%) | Normal morphology (%) |
|---|---|---|---|---|---|---|---|---|---|
| **32** | 32.5 [29.3-37.8] | 7.6 [7.6-7.8] | 4.1 [2.8-5.4] | 43.5 [32.0-69.6] | 187.7 [112.1-320.1] | 52.5 [46.5-59.5] | 10.0 [6.0-12.8] | 38.0 [31.3-41.0] | 4.5 [3.0-6.0] |
