# Supplementary material for: Effects of common Gram-negative pathogens causing male genitourinary-tract infections on human sperm functions
Source: Sci Rep. 2021 Sep 28;11:19177. doi: 10.1038/s41598-021-98710-5 (PMC8478950; doi:10.1038/s41598-021-98710-5)
Supplement: Supplementary file 4 — Supplementary Table 2. [file 41598_2021_98710_MOESM4_ESM.pdf]

EFFECTS OF COMMON GRAM-NEGATIVE PATHOGENS CAUSING MALE GENITOURINARY-TRACT INFECTIONS ON HUMAN SPERM FUNCTIONS

Sara Marchiani, Ilaria Baccani, Lara Tamburrino, Giorgio Mattiuz, Sabrina Nicolò, Chiara Bonaiuto, Carmen Panico, Linda Vignozzi, Alberto Antonelli, Gian Maria Rossolini, Maria Torcia, Elisabetta Baldi

Supplemental Table 2. Selected ATCC strains and relative source of isolation

Reference strain number (ATCC)	Species	Sequence Type (ST)	Source	Human/Animal
27853	*P. aeruginosa*	155	Blood	Human
13883	*K. pneumoniae*	3	unknown	unknown
700603	*K. quasipneumoniae*	489	Urine	Human
25922	*E. coli*	73	unknown	Human
35218	*E. coli*	127	Canine	Human
13047	*E. cloacae*	1	Spinal fluid	Human
13048	*K. aerogenes*	134	Sputum	Human
