# Supplementary material for: Effects of common Gram-negative pathogens causing male genitourinary-tract infections on human sperm functions
Source: Sci Rep. 2021 Sep 28;11:19177. doi: 10.1038/s41598-021-98710-5 (PMC8478950; doi:10.1038/s41598-021-98710-5)

EFFECTS OF COMMON GRAM-NEGATIVE PATHOGENS CAUSING MALE GENITOURINARY-TRACT INFECTIONS ON HUMAN SPERM FUNCTIONS

Sara Marchiani, Ilaria Baccani, Lara Tamburrino, Giorgio Mattiuz, Sabrina Nicolò, Chiara Bonaiuto, Carmen Panico, Linda Vignozzi, Alberto Antonelli, Gian Maria Rossolini, Maria Torcia, Elisabetta Baldi

Supplemental Figure S1. Frames from a video of a semen sample in vitro incubated with *E. cloacae* ATCC 13047 for 3 hours at 37°C, 5%CO_2. Adhesion of bacteria to the tail is evident in many spermatozoa (Nikon Eclipse Ci phase contrast microscope with heated stage, Leica MC170 HD camera, resolution 5 Megapixel).

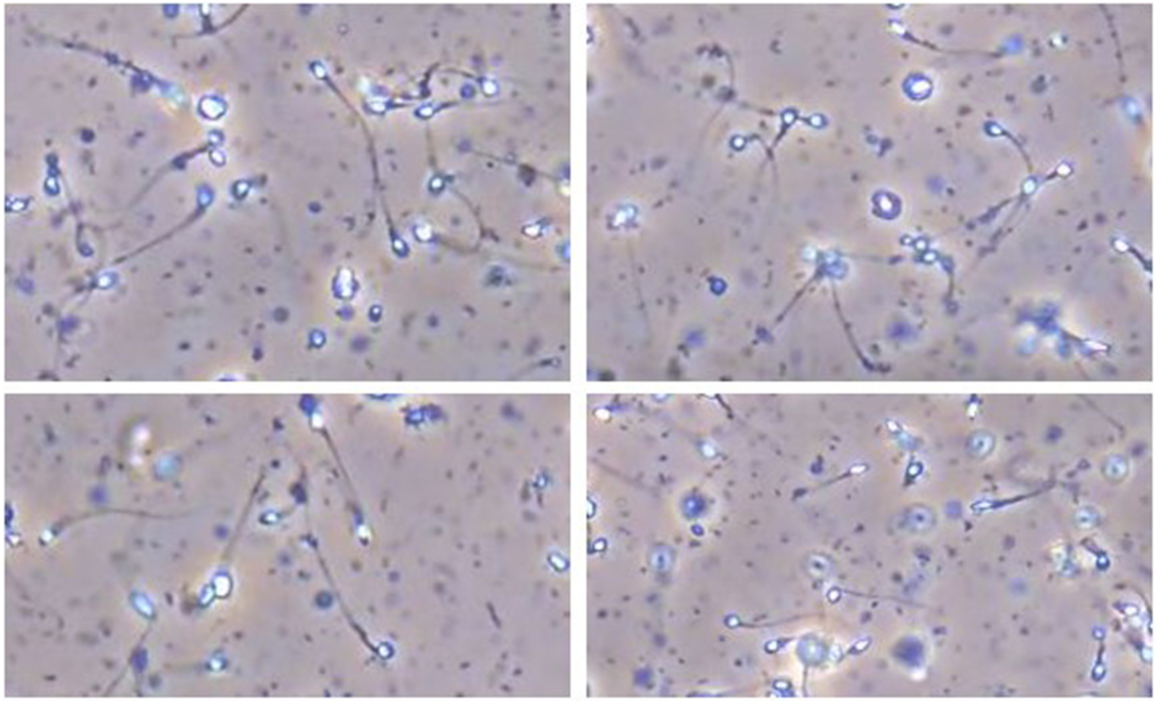

Supplement: Supplementary file 5 — Supplementary Figure 1. [file 41598_2021_98710_MOESM5_ESM.pdf]
